# Aberrant ERK 1/2 complex activation and localization in scrapie-infected GT1-1 cells

**DOI:** 10.1186/1750-1326-5-29

**Published:** 2010-08-09

**Authors:** Alessandro Didonna, Giuseppe Legname

**Affiliations:** 1 Laboratory of Prion Biology, Neurobiology Sector, Scuola Internazionale Superiore di Studi Avanzati (SISSA), via Bonomea 265, I-34136 Trieste, Italy; 2 ELETTRA Laboratory, Sincrotrone Trieste S.C.p.A., S.S. 14 Km 163.5, I-34149 Basovizza (TS), Trieste, Italy; 3 Italian Institute of Technology, SISSA Unit, via Bonomea 265, I-34136 Trieste, Italy

## Abstract

**Background:**

Fatal neurodegenerative disorders such as Creutzfeldt-Jakob and Gerstmann-Sträussler-Scheinker diseases in humans, scrapie and bovine spongiform encephalopathy in animals, are characterized by the accumulation in the brain of a pathological form of the prion protein (PrP) denominated PrP^Sc^. The latter derives from the host cellular form, PrP^C^, through a process whereby portions of its α-helical and coil structures are refolded into β-sheet structures.

**Results:**

In this work, the widely known *in vitro *model of prion replication, hypothalamic GT1-1 cell line, was used to investigate cellular and molecular responses to prion infection. The MAP kinase cascade was dissected to assess the phosphorylation levels of src, MEK 1/2 and ERK 1/2 signaling molecules, both before and after prion infection. Our findings suggest that prion replication leads to a hyper-activation of this pathway. Biochemical analysis was complemented with immunofluorescence studies to map the localization of the ERK complex within the different cellular compartments. We showed how the ERK complex relocates in the cytosol upon prion infection. We correlated these findings with an impairment of cell growth in prion-infected GT1-1 cells as probed by MTT assay. Furthermore, given the persistent urgency in finding compounds able to cure prion infected cells, we tested the effects on the ERK cascade of two molecules known to block prion replication *in vitro*, quinacrine and Fab D18. We were able to show that while these two compounds possess similar effects in curing prion infection, they affect the MAP kinase cascade differently.

**Conclusions:**

Taken together, our results help shed light on the molecular events involved in neurodegeneration and neuronal loss in prion infection and replication. In particular, the combination of chronic activation and aberrant localization of the ERK complex may lead to a lack of essential neuroprotective and survival factors. Interestingly, these data seem to define some common traits with other neurodegenerative disorders such as, for example, Alzheimer's disease.

## Background

Transmissible spongiform encephalopathies (TSE) or prion diseases, such as Creutzfeldt-Jakob disease, Gerstmann-Sträussler-Scheinker syndrome and fatal familial insomnia in humans, bovine spongiform encephalopathy and scrapie in animals, are a group of incurable neurodegenerative disorders. TSE can manifest as spontaneous, inherited and infectious maladies. These diseases are caused by the accumulation of prions in the central nervous system (CNS).

Prions are novel infectious agents composed solely of a pathological isoform of the prion protein (PrP) PrP^Sc^, derived from the host-encoded, cellular form of PrP, PrP^C ^[1]. PrP^Sc ^accumulation is driven by a conversion event in which α-helix and random coiled structures are refolded into β-sheets [2]. The PrP^C ^molecule is a membrane glycoprotein highly expressed in neurons and is linked to the outer leaflet of neuronal membranes via glycosylphosphatidylinositol moiety localized in cholesterol-rich domains called "rafts". Despite PrP^C ^being conserved amongst mammals, its function is still ambiguous and defining the cellular processes involved in prion disease remains one of the main challenges in Prion Biology.

While PrP-null mice (*Prnp*^0/0^) do not show gross phenotypic abnormalities [3], analysis of *in vitro *models of primary cells derived from *Prnp*^0/0 ^revealed a dysmetabolism of copper and an increased susceptibility to oxidative stress [4], suggesting the involvement of PrP^C ^in redox homeostasis and in copper uptake within the cell. Additional studies revealed a role for PrP^C ^in cellular adhesion, showing that PrP interacts with neural cell adhesion molecules (N-CAMs), laminin and laminin receptor [5,6].

Moreover, PrP^C ^can regulate neurite outgrowth [7] and neuroprotection [8] in primary cultures of neurons. These two latter effects rely on the interaction of PrP with stress-inducible protein 1 (ST1) and seem to be mediated by distinct signaling pathways [9]. These different lines of experimental evidence suggest that PrP^C ^may also transduce signals from the membrane to the nucleus. It has been established that PrP^C ^mediates activation of cAMP/protein kinase A (PKA) in retinal tissue [10] and activation of protein kinase C (PKC) in embryonic rat hippocampal neurons [7]. Moreover, PrP^C ^seems to be involved in regulation of calcium-mediated cellular events [11]; in *Prnp*^0/0 ^mice a decrease of calcium influx via VGCC was found, suggesting a functional interaction with calcium channels on the cell membrane [12]. Furthermore, in prion-infected neuroblastoma cells a decrease in receptor-mediated calcium responses was observed [13]. In addition, PrP^C ^has been recently identified as an amyloid-β-oligomer receptor and it appears to mediate impairment of synaptic plasticity in Alzheimer's disease (AD) [14]. Among others, also mitogen-activated protein kinases (MAPKs) pathways seem to be regulated by PrP^C^. These signaling cascades are strongly conserved in eukaryotic cells and modulate molecular events involved in cell differentiation, proliferation and apoptosis, and in gene expression and inflammation [15] processes, whose deregulation plays a crucial role in neurodegenerative diseases such as TSE. All these cascades are composed of three distinct modules: MAP kinases, MAPK kinases and MAPKK kinases. In mammals, four sets of MAPKs are expressed: extracellular signal-related kinases (ERK), Jun-amino terminal kinases (JNK), p38 proteins and ERK5 [16]. These cascades have been observed in several cellular models linked to PrP biology. By mimicking the binding of a cellular interactor by antibody mediated cross-linking, a PrP-dependent fyn activation was observed in 1C11 cells [17]. Using a similar approach, ERK1/2 pathway activation was observed in GT1-7 cells [18]. In monocytes, ERK activation was obtained using PrP fusion protein with a Fc domain [19]. The treatment of hippocampal neurons in culture with either hop/STI1 or hop/STI1230-245 also led to ERK activation [9]. The same results were obtained developing retinal tissues using binding molecules for PrP^C ^[10]. Hyper-activation of several MAP kinases was observed in hamster brains infected with 263 K scrapie strain [20], and elevated levels of the src family kinases were found in an *in vitro *model and two different *in vivo *models of prion disease [21]. Recent studies have linked the increased level of phosphorylation of the MAP kinases pathway to a neuroprotective and antiapoptotic effect towards toxicity of PrP^Sc ^[22]. In contrast, in a model of prion disease using prion peptide 106-126, the overstimulation of ERK pathway was correlated with oxidative injury in GT1 cells throughout ROS production [23]. According to this model oxidative stress plays a central role in neurodegenerative diseases [24].

In this study we used a neuronal cell model, GT1-1 hypothalamic cell line [25], known to support a sustained prion replication. To investigate cell response to prion infection, we focused on MAP kinases. In particular we dissected the ERK pathway, analyzing the levels of phosphorylation of key molecules such as src, MEK and ERK before and after prion infection, both *de novo *and chronic. We then mapped the distribution of phospho-ERK within the different cellular compartments in infected and uninfected cells. For the first time we showed how prion infection has the capacity of altering the levels of activation and the cellular localization of the ERK complex, affecting cell proliferation. In addition, cells were treated with Fab D18 [26] and quinacrine [27], two molecules known to cure prion infection in cell culture, and assessed for the extent of phosphorylation activation. Our findings show how the two compounds exert different effects on the ERK pathway, suggesting that the two molecules may interfere with prion infection via different pathways.

## Results

### Scrapie infection affects MAP kinases levels in GT1 cells

In recent years several cell systems permissive to the replication of different mouse-adapted prion strains have been developed [28]. They represent a suitable tool for studying the molecular basis of prion-induced neurodegeneration; their simplicity, in fact, allows to analyze the events occurring during prion pathogenesis much better than *in vivo *models.

**Figure 1:**
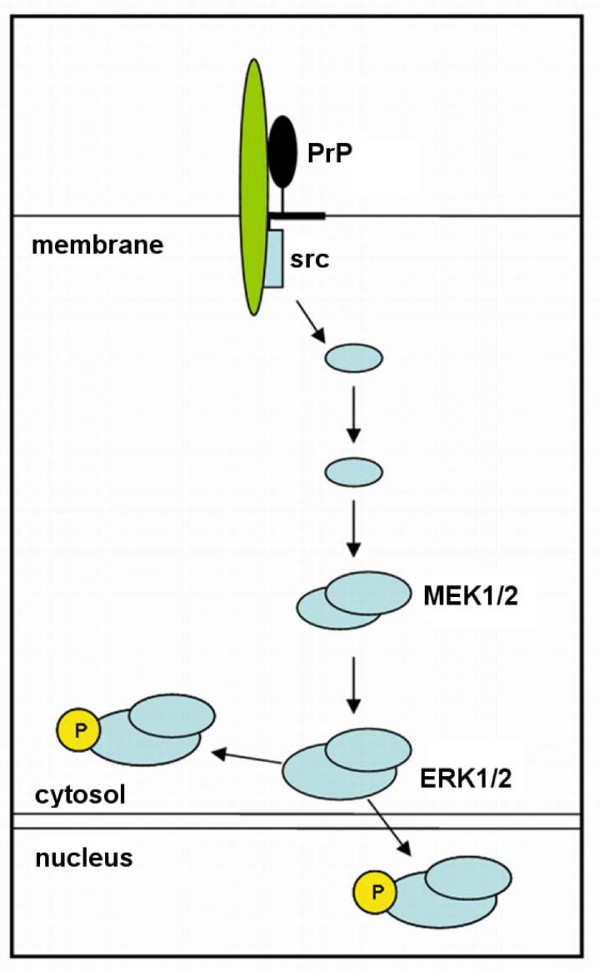
**Schematic representation of ERK pathway**. PrP^C ^on the cellular surface assembles a signaling platform with src family proteins. Upon activation, this molecular complex modulates the activity of ERK cascade. Src proteins activate MAP/ERK Kinase 1/2 (MEK1/2). The active forms of MEK1/2 complex can phosphorylize the Extracellular signal-Regulate Kinase 1/2 (ERK1/2) that either enters the nucleus and starts gene expression, or remains within the cytosol.

To investigate the response of neurons towards prion infection we chose, as a model of prion replication, an immortalized murine cell line derived from hypothalamic cells either uninfected or chronically infected with RML prion strain (GT1 cells and ScGT1 cells respectively, hereafter). In order to elucidate some of the mechanisms leading to neuronal loss we focused on MAP Kinase pathways, given their pivotal role in cell growth and survival. We dissected one of the pathways, the ERK cascade, and, in particular, we analyzed the src family, MEK and ERK kinases. The src family is composed of proteins located at the early stages of the ERK cascade, next to the inner leaflet of the cellular membrane. The MEK1/2 protein complex is the physiological activator of ERK1/2, that, in turn, upon activation, can either enter the nucleus and start the gene expression process, or be retained in the cytoplasm to activate other signaling molecules (Fig. 1).

**Figure 2:**
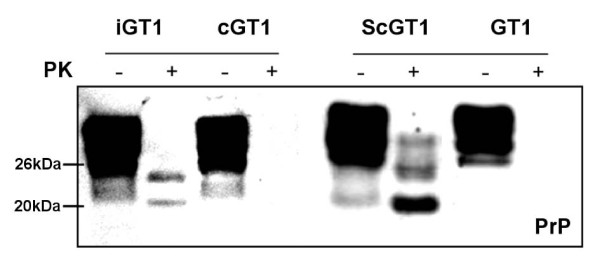
**Chronic and *de novo *infection of GT1 cell line**. GT1 cells were infected according to the procedures described in the Materials and Methods section. Regarding *de novo *infected GT1 cells (iGT1) the presence of PrP^Sc ^was tested at the fourth passage. PrP^Sc ^was detected by PK digestion assay (PK+ lanes) and the signal revealed using Western blot technique. Approximately 20 μg of total protein were loaded as control (PK-lanes).

**Figure 3:**
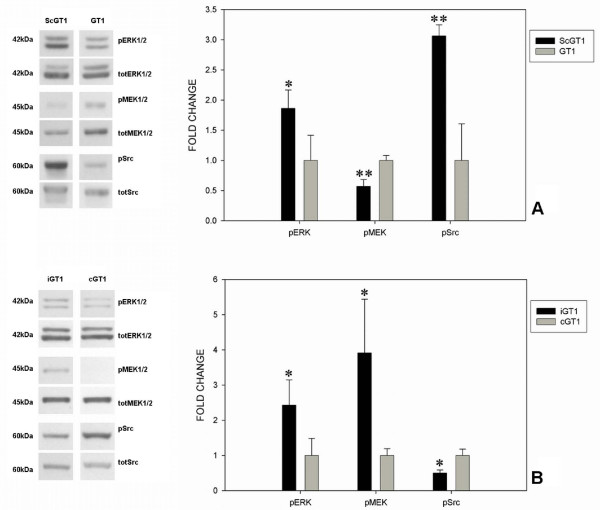
**Analysis of ERK pathway in chronic and *de novo *infected GT1 cells**. Approximately 20 μg of total proteins were resolved on 12% polyacrylamide gels to detect the levels of phosphorylated forms of ERK 1/2, MEK 1/2 and src family proteins. The total amount of the same proteins was determined as a control. Prions induced a general hyper-activation of ERK cascade in chronic infected GT1 cells (ScGT1) compared to uninfected cells (GT1). Levels of phospho-ERK and phospho-src proteins increased upon prion infection, whilst a decrease was found in levels of phospho-MEK (panel A). In *de novo *infected cells (iGT1) the levels of phospho-ERK and phospho-MEK were increased compared to mock-infected ones (cGT1), whilst a decrease of phospho-src was detected upon prion infection (panel B). Statistics were performed using Student's T-test on a set of three independent experiments; data were normalized on the total amount of proteins. * P < 0.05, ** P < 0.01 versus uninfected cells.

Total cell extracts from GT1, both uninfected and infected (Fig. 2), were tested for phosphorylation levels of the kinases. In ScGT1 cells, scrapie infection led to a general overstimulation of the ERK cascade. Immunoblot revealed a statistically significant increase of phospho-ERK levels in ScGT1 compared to uninfected cells, whilst a decrease was detected in the level of phospo-MEK upon prion infection (Fig. 3A). Instead, the densitometric analysis between the levels of phospho-src in infected and uninfected cells revealed an increased phospho-src signal in ScGT1 (Fig. 3A).

The activation of ERK pathway was also tested in *de novo *infected cells. GT1 cells were infected as described in the Materials and Methods section, and tested at the fourth passage upon infection (Fig. 1). *De novo *infected GT1 cells (iGT1) showed a statistically significant increase of phospho-ERK and phospho-MEK levels compared to mock infected cells (cGT1) (Fig. 3B). On the contrary, the levels of phospho-src were found strongly decreased in iGT1 cells in compared to cGT1 cells (Fig. 3B).

### Altered phosphorylation levels upon Fab D18 and quinacrine treatments

To date an effective cure for prion disease has not been found, although several compounds are known to block prion replication at least *in vitro*. Considering those as models for the development of new therapeutic strategies, we studied the actions of two of them, Fab D18 and quinacrine, on the ERK pathway in order to evaluate their side effects on cell physiology.

**Figure 4:**
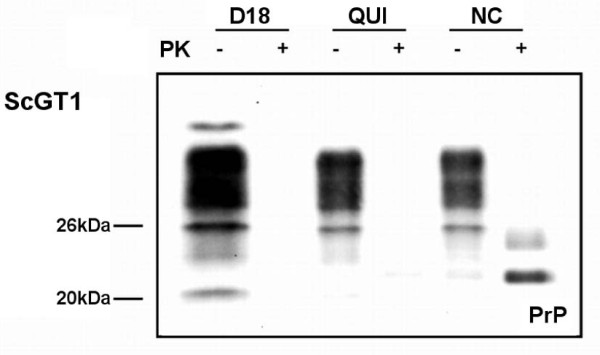
**Effect of D18 and quinacrine treatments on PrP^Sc ^levels in ScGT1 cells**. RML infected GT1 were treated for 6 days with 1 μM quinacrine and 50 nM D18. Cell lysates were digested with proteinase K (PK, PK+ lanes) as described in Materials and Methods and PrP^Sc ^content was detected by Western blot. Twenty μg of total proteins (PK-lanes) were loaded as control. Noticeably, PK-resistant PrP was completely cleared in GT1 cells.

We treated both infected and uninfected cells for six days with Fab D18 at the final concentration of 50 nM and with quinacrine at the concentration of 1 μM, according to tested concentrations available in the literature [26,27]. The PrP^Sc ^signal was no longer present in ScGT1 cells after the Fab and drug treatments as ascertained by Western blot (Fig. 4). Next, we analyzed the levels of phosphorylation of src, MEK and ERK kinases, both before and after the treatments.

**Figure 5:**
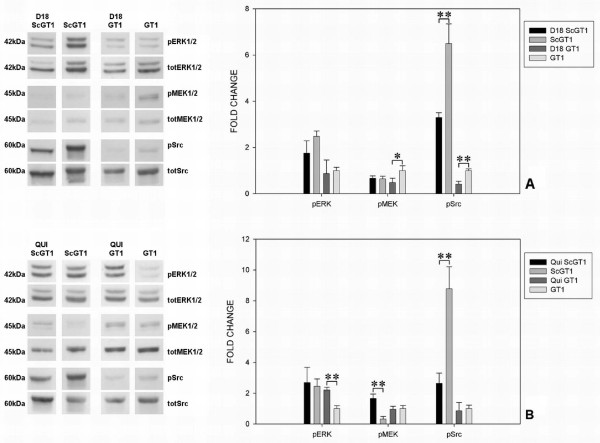
**Effect of D18 and quinacrine treatment on ERK pathway in GT1 cells**. Infected and uninfected GT1 cells were treated with Fab D18 (50 nM) or quinacrine (1 μM) for 6 days; approximately 20 μg of total proteins were loaded onto 12% polyacrylamide gel and assessed for the levels of the phosphorylated forms of ERK 1/2, MEK 1/2 and the src family proteins. The total amount of the same proteins was determined as a control. Fab D18 did not appear to induce any significant changes in the levels of phospho-ERK both in infected and uninfected GT1, whilst decreased levels of phospho-MEK were detected in GT1 cells upon D18 treatment (panel A). Regarding phospho-src proteins, increased levels were found in treated GT1 compared to untreated cells, both infected and uninfected (panel A). On the contrary, quinacrine treatment induces a significant hyper-stimulation of the ERK protein in GT1 cells and even increases the levels of the phospho-MEK in ScGT1 (panel B). Moreover, a strong decrease on phospho-src levels were found in infected GT1 upon drug treatment (panel B). Statistics were performed using Student's T-test on a set of three independent experiments; data were normalized on the total amount of proteins. * P < 0.05, ** P < 0.01 versus untreated controls, both for infected and uninfected cells.

Following Fab D18 treatment, no significant variations of phospho-ERK levels were found either in ScGT1 or GT1 cells, whereas a significant decrease in D18 treated GT1 was found with regards to phospho-MEK levels (Fig. 5A). Concerning the src family, incubation with the antibody seemed to significantly decrease the level of phospho-src, both in infected and uninfected GT1 cells (Fig. 5A). Upon quinacrine treatment, the levels of phospho-ERK were unaffected in ScGT1 cells, whilst drug incubation increased the levels of phospho-ERK in non-infected GT1 cells (Fig. 5B). With regards to phospho-MEK levels, quinacrine increased its levels in ScGT1 cells, but not in GT1 (Fig. 5B). Considering the src family activation, levels of phospho-src were strongly decreased in ScGT1 upon quinacrine treatment, whilst the drug was found to have no effect on src family activation in GT1 cells (Fig. 5B).

### Cellular localization of phospho-ERK

When analyzing the action of the ERK pathway, it can be noted that besides being biochemically regulated through the phosphorylation of its kinases, the localization of the latter within the different cellular compartments plays a pivotal role in establishing the correct signaling. Upon phosphorylation, in fact, the ERK complex can either be found in the nucleus, or selectively retained within the cytosol. We therefore mapped the cellular distribution of phospho-ERK by means of immunocytofluorescence, aiming at further elucidating the various mechanisms occurring in prion-infected cells.

Both chronically infected, and uninfected, D18 and quinacrine treated and untreated cell lines, were fixed and stained according to the procedures in the Materials and Methods section, and phospho-ERK localization was investigated by confocal microscopy.

**Figure 6:**
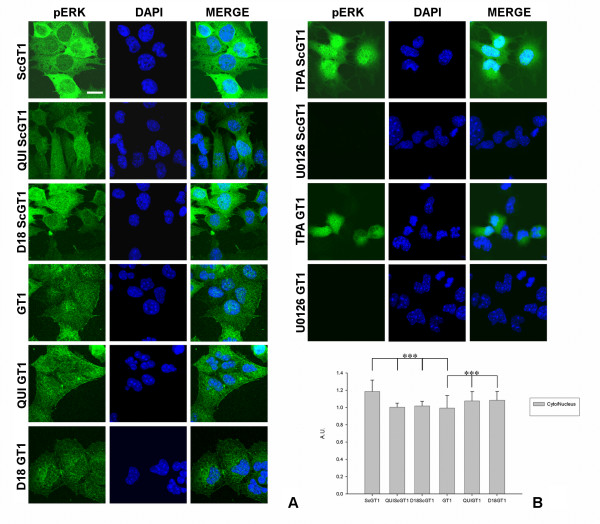
**Immunolocalization of phospho-ERK 1/2 within GT1 cells**. Infected and uninfected GT1 cells, both D18 and quinacrine treated and untreated, were fixed and immunostained with an antibody specific for the phosphorylated (Thr202/Tyr204) form of ERK 1/2 (in green); nuclei were counterstained with DAPI (in blue). Merged images are shown on the left. In ScGT1 phospho-ERK appears prevalently localized within the cytoplasm, whilst in GT1 cells the phospho-ERK staining is equally distributed between the cytosolic and the nuclear compartments (panel A). The treatments with both Fab D18 and quinacrine induce the active form of ERK to localize preferentially within the nuclear compartment in ScGT1, but have opposite effects on the phospho-ERK distribution in uninfected GT1 (panel A). As positive control, cells were treated with TPA (12-O-Tetradecanoylphorbol-13-Acetate) diluted in DMSO at the concentration of 200 nM for 30 minutes. As negative control, U0126 (10 μM in DMSO) was used, treating cells for 1 hour (panel A). Images are representative of at least three independent experiments of immunostaining. Scale bars, 20 μm. The quantitative analysis was carried out measuring the average intensity of fluorescence in a region of interest (ROI) of 144 pixels, both in the nuclear and cytoplasmatic regions. The histograms (panel B) show the ratio between the average intensity of fluorescence of phospho-ERK signal in the cytosol, over the average intensity of the same signal in the nucleus. More than 100 cells were analyzed for each condition. Statistics were performed using Student's T-test on a set of three independent experiments; *** P < 0.001.

The pattern of phospho-ERK distribution for ScGT1 consisted in a granular dispersion of punctuate labeling localized within the cytoplasm, more pronounced in the perinuclear area, whilst in GT1 cells phospho-ERK staining was equally divided between the cytosol and the nucleus (Fig. 6A). The treatment with both Fab D18 and quinacrine seemed to increase the fraction of phospho-ERK localized in the nuclear compartment in treated ScGT1 (Fig. 6A), while an opposite effect was detected in treated uninfected GT1cells (Fig. 6A). Statistical analysis was carried out as described in the Materials and Methods section (Fig. 6B).

### Prion infection affects cell proliferation

**Figure 7:**
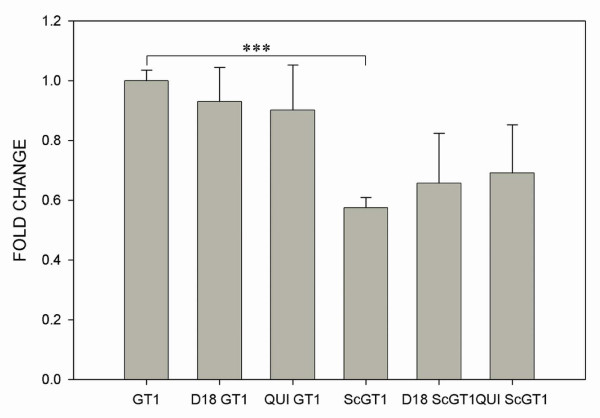
**Scrapie infection affects cell proliferation**. Cell proliferation was evaluated in ScGT1 and GT1 cells, pre-treated for 6 days with quinacrine (1 μM) and Fab D18 (50 nM), over 4 days by means of MTT assay. Prion infection inhibits cell proliferation and both treatments seem to have no effect on it. Cell growth rate was calculated as described in Materials and Methods. All data come from three independent experiments performed each one in 5 replicates; they are expressed as mean value ± SD. Statistics were performed using Student's T-test, *** P < 0.001.

As ERK cascade is involved in cell growth and survival; in order to correlate the molecular findings about aberrant signaling with defined physiologic effects, the cell proliferation for both ScGT1 and GT1 was monitored before and after the treatment with Fab D18 and quinacrine by means of MTT assay. Without treatment a 40% lower growth rate for the ScGT1 cells was found compared to uninfected cells (Fig. 7). Following treatment with Fab D18 (50 nM) and quinacrine (1 μM), cells were tested for proliferation, again in the presence of the two compounds at the same concentrations. MTT assay did not show any significant effects on cell growth of both ScGT1 and GT1 after the two treatments (Fig. 7).

## Discussion

### Prion infection hyper-stimulates ERK cascade

Whilst the pathological mechanism of prion diseases remains elusive, many lines of evidence indicate that prion infection may dramatically alter the physiology of the cell. Therefore, the focus of our work consisted in studying molecular signaling molecules in both healthy and prion infected cells. We employed a well-known neuronal model, the murine immortalized hypothalamic GT1 cell line, able to sustain both *de novo *and chronic prion infection.

First, we analyzed the effect of prion infection in chronic infected cells. In particular, we assessed the ERK cascade, a pathway conserved throughout all classes of mammals and involved in processes such as cell growth, neuronal survival, axonal outgrowth and long-term plasticity [29,30]. The ERK pathway has been proposed as a possible target for TSE therapy since several tyrosine kinase inhibitors, such as STI571 and imatinib mesylate, have recently been shown to induce a complete clearance of PrP^Sc ^in ScN2a cells and a decrease of its levels in scrapie-infected mouse spleens [31,32]. In addition, inhibitors of the MEK 1/2 complex have proven to be efficient in clearing PrP^Sc ^from ScGT1 cells [33]. Focusing on the three key nodes of ERK pathway, the src family, MEK1/2 and ERK1/2 complexes, we found that prion replication leads to a general hyper-activation of this cascade in GT1-1 cells. Interestingly, although we detected an increased level of phosphorylation for the ERK complex, we also found a lower level of activation of the MEK complex. We can speculate that this apparent paradox involves a different action of some phosphatases on ERK1/2 upon chronic prion infection.

We also examined the activation state of the pathway in *de novo *infected cells to assess the role of cloning selection in ERK signaling. We confirmed the activation of the pathway in GT1 cells upon prion infection, though the levels of phosphorylated proteins were not the same as for chronic infected cells. In this case we were able to detect increased levels for both phospho-MEK and phospho-ERK complexes in newly infected cells, but not for the src family. Most likely, since the antibody we used to detect src proteins recognizes all the 9 members of the family, the decrease in phospho-src kinases may be explained considering the contributions of all the members of the family to the signal detected by Western blotting. In response to acute infection some members of the src family may undergo a massive negative regulation in terms of phosphorylation. While the src family member fyn seems to be the tyrosine kinase directly involved in the activation of the ERK pathway via PrP [17], other four members of the family (src, yes, lck and lyn) are expressed within the CNS and act on different substrates [34]. It has been shown that the tyrosine kinase inhibitor STI571 is able to block prion replication in scrapie-infected cells, promoting PrP^Sc ^clearance throughout the lysosomal pathway [31]. Thus, it seems that the aberrant activation of src family proteins in chronically infected cells results in an impaired function of the lysosomal compartment, leading to PrP^Sc ^accumulation. So, the negative regulation undergone by some members of the src family, when GT1 cells were exposed to prions in acute fashion, may be interpreted as a feedback response of the cell to induce PrP^Sc ^catalysis. The self-propagating nature of prions and the foundation of a chronic infection in the long term, may lead to the loss of this cellular response, resulting in the aberrant activation of src kinases found in the ScGT1 cells.

### ERK retention within the cytoplasm: a possible cause for neurodegeneration?

Taken together, these data may unveil at least one possible molecular mechanism responsible for neuronal loss upon prion infection. Besides the archetypical functions of the ERK cascade in cell survival and proliferation, recently a novel role in regulating cell death has emerged in neurodegenerative diseases. Phospho-ERK aggregates were found in the *substantia nigra *of Parkinson's disease patients [35]. Elevated levels of phospho-ERK were also revealed in brain extracts of AD patients, but not in control individuals [36]. Moreover, a chronic activation of the ERK complex was detected in organotypic hippocampal slides from animal models for AD overexpressing Aβ [37]. In addition to the chronic activation of ERK, its localization within the cell seems to also play a crucial role in initiating neurodegenerative processes [38]. An intriguing scenario in which an aberrant activation of ERK, in kinetic and spatial terms, is linked to cell death processes seems to fit with our findings in GT1 cells, expanding our understanding of prion diseases. Both chronically and newly infected ScGT1 cells showed a persistent activation of ERK. Since prion conversion requires direct interaction between PrP^C ^and PrP^Sc ^most likely in the amyloidogenic domain (AGAAAAGA) of PrP (amino acids 109-122) [39], in ScGT1 cells prion replication may lead to aberrant signaling. Upon infection, the formation of multimeric structures of PrP, such as oligomers, may trigger the chronic activation of the ERK pathway. Immunocytochemistry data seem to provide support to our model. A different compartmentalization of phospho-ERK was described in infected and non-infected GT1 cells. While the active form of ERK seemed to be efficiently translocated in the nuclear compartment of uninfected cells, upon prion infection phospho-ERK appeared to be preferentially retained in the cytoplasm. Moreover, we showed how cell growth rate was lower in ScGT1 cells compared to uninfected cells. Tentatively, the impairment of nuclear translocation in infected cells may be correlated with the failed transcription of neuroprotective factors essential for cell survival and could explain the differences found in cell proliferation. In addition, upon prion infection in GT1 cells, a hyper-phosphorylation of the S6 ribosomal protein has been described [40]. Since S6 protein is a target for the ERK complex, further studies are needed to assess whether the preferential localization of the active form of ERK in the cytoplasm of scrapie-infected ScGT1 can be also associated to the phosphorylation of S6 protein. The lack of gross pathological phenotype in ScGT1 could be explained by considering GT1 as active proliferating cells. Cell divisions may dilute the amount of PrP^Sc ^among daughter cells, minimizing marked pathological effects on cell physiology. In post-mitotic neuronal cells of the CNS, PrP^Sc ^can accumulate and ultimately lead to neurodegeneration. This could explain why subpopulations of infected ScGT1 show reduced viability, with signs of neurodegeneration and vacuolation [41]. For some cells, where prion conversion rate is higher, the ERK pathway could be hyper-stimulated and detectable cellular damages may occur.

### Fab D18 and quinacrine treatments differently affect the ERK pathway

The second aim of our study was to assess the effects on the ERK cascade induced by two molecules known to block prions. In recent years, much effort has been devoted to finding a molecule that would halt prion propagation. To date, two classes of compounds yielded promising results: small chemicals such as acridines [27] and antibodies, or antibody fragments, such as Fab D18 [26]. In our study, both infected and uninfected cells were treated with the two compounds to assess the effect on the activation of ERK pathway. Two main datasets emerged from our study, which revealed different effects on the ERK pathway when comparing infected and uninfected cells. Whilst the epitope for Fab D18 on PrP is well known [42], the binding surface on PrP for quinacrine is still debated. Moreover, the affinity of Fab D18 for PrP is much higher than for quinacrine; the dissociation constant for the quinacrine-PrP^C ^complex is 4.6 mM [43], whereas for the Fab D18-PrP^C ^complex is 1.6 nM [26]. Thus, the two compounds might act through different mechanisms. In particular quinacrine may interfere with prion replication through a process that does not require its binding to PrP^C^, since the half-maximal PrP^Sc ^inhibition occurs at effective concentrations [EC(50)] in the micromolar range [27]. Recent studies, for example, have shown acridines binding directly to PrP^Sc ^[44]. These data may also explain the differences found in terms of ERK pathway activation between infected and uninfected cells after treatment with the two compounds. Since Fab D18 carries on its inhibitory activity principally through binding to PrP^C ^and thus preventing its interaction with PrP^Sc^, the effects on cell signaling are similar both in ScGT1 and GT1 cells as they both share PrP^C ^expression. On the contrary, quinacrine has a low affinity for PrP^C ^and seems to block prion replication by binding and disrupting PrP^Sc ^fibrils [45]. Large aggregates of PrP^Sc ^are distinctive traits of infected cells, so it is reasonable that the effects of quinacrine treatment on the ERK pathway in infected and uninfected cells are very different. Alternatively, quinacrine might bind to protein(s) involved in prion replication, or may alter the biochemical environment necessary for template refolding. For many years the so-called "protein X" has been hypothesized to play an essential role in prion replication, a protein, maybe a chaperon, interacting with the PrP^C^-PrP^Sc ^complex and facilitating conversion. The binding of quinacrine to a key site of this putative molecule may alter its normal function in GT1 cells and interfere with the prion conversion process.

In addition, even though quinacrine and Fab D18 seem to maintain the physiological distribution of phospho-ERK in GT1 cells, both of them failed to revert ERK phosphorylation to physiological levels upon treatment of prion infected ScGT1. They also failed in rescuing the impaired cell proliferation detected in ScGT1 cells. These data could be explained considering that prion infection may alter some fundamental mechanism involved in cell division irreversibly. As an alternative, it could be hypothesized that the two treatments do not block prion replication completely, but just decrease it below the sensitivity of detection techniques. Moreover, the ability of the two molecules to alter the localization and the activation levels of ERK cascade in uninfected cells should be taken into consideration as a significant side effect in any therapeutic approach to prion disease.

The MEK inhibitor U0126 was also tested for its cell proliferation rescuing capacity in ScGT1 cells (see additional file 1). No significant differences in terms of cell growth were detected after 3 days of treatment, using a concentration of drug that completely inhibited the ERK complex phosphorylation (10 μM) (see additional file 1). Thus, identifying a pharmacological treatment that not only clears prion infection, but also does not carry any apparent side effect on healthy cells, still remains a major challenge.

## Conclusions

In this study we investigated the role of prion infection in ERK cascade signaling using a well-known neuronal model of prion replication. For the first time we were able to show a correlation between the aberrant activation of the ERK1/2 complex, its localization within the cytosolic compartment, and a lower growth rate in infected GT1 cells. Our findings indicate a possible mechanism of neurodegeneration that might explain the neuronal loss expressed upon prion disease onset. In addition, we tested two prion-curing compounds for their actions on the ERK pathway. The different responses observed may be correlated to different mechanisms of action.

## Materials and methods

### Cell lines and cell culture

GT1 and ScGT1 cells were maintained in Dulbecco's Modified Eagle's Medium with 4.5 g/L glucose (DMEM) (GIBCO/Invitrogen), supplemented with 10% v/v fetal bovine serum (GIBCO/Invitrogen) and antibiotics (100 IU/mL penicillin and 100 mg/mL streptomycin) at 37°C in a humidified atmosphere with 5% CO_2_. All cell lines were kindly provided by Dr. P. Mellon (The Salk Institute, La Jolla, CA, USA). Scrapie cells were chronically infected with Rocky Mountain Lab (RML) prion strain according to already published procedures.

### *De novo *prion infection

ScGT1 cells were grown at confluence and fixed in paraformaldehyde 4% in PBS for 20 min at room temperature (RT). Then, the cell layer was washed in PBS three times and permeabilized with TRITON XT-100 0.1% in PBS for 15 min. After further washings with PBS, GT1 cells were cultured on this "infective layer" for 1 week, refreshing the medium on the third day. Then cells were split 1:3 for three times and tested at the fourth passage. Cells were grown on fixed GT1 cells as control.

### Antibodies

The monoclonal antibodies against the phosphorylated and non-phosphorylated forms of the src family [Src Family Antibody #2109 and phospho-Src Family (Tyr416) antibody #2101), MEK1/2 (MEK1/2 Antibody #9122 and phospho-MEK1/2 (Ser217/221) (41G9) rabbit mAb #9154] and ERK 1/2 [p42 MAP Kinase (3A7) mouse mAb #9107 and phospho-p44/42 Map Kinase (Thr202/Tyr204) Ab #9101] were purchased from Cell Signaling Technology. The Fab D18 fragment raised against PrP was purchased from InPro Biotech.

### Preparation of cytosolic extracts

Cells were treated for 6 days with Fab D18 (50 nM), or quinacrine (Fluka), which had been dissolved in PBS (1 μM). The medium was refreshed after 3 days. Cells were then washed twice with cold PBS 1× (GIBCO/Invitrogen) and incubated for 10 min on ice, in lysis buffer [50 mM Tris-HCl (pH 7.4) 150 mM NaCl, 1% Triton X-100, 2 mM Na_3_VO_4 _and a mixture of protease inhibitors (Roche)]. The cell extracts were then centrifuged at 2,300 g for 5 min. The supernatant was stored at -80°C before being used. Total protein concentration was determined using the bicinchoninic acid assay (Pierce).

### Proteinase K digestion assay

Cells were washed twice with cold PBS 1× (GIBCO/Invitrogen) and lysed with lysis buffer (10 mM Tris-HCl pH 8.0, 150 mM NaCl, 0.5% nonidet P-40 substitute, 0.5% deoxycholic acid sodium salt) and pelleted by centrifugation at 2,300 g for 5 min. The supernatant was collected and the total protein concentration measured using bicinchoninic acid assay (Pierce). For the assay, 250 μg of protein was treated with 5 μg proteinase K (Roche, ratio protein:protease 50:1) for 1 hour at 37°C. Digestion was stopped by the addition of phenylmethyl sulphonyl fluoride to a final concentration of 2 mM. The PrP was precipitated by ultracentrifugation at 100,000 g (Optima TL, Beckman) for 1 hour at 4°C. After centrifugation, the supernatant was discarded and the pellet resuspended in loading buffer 1X, before loading onto a 12% SDS-PAGE. Samples were electroblotted onto membranes of polyvinylidene fluoride (PVDF). PVDF membranes were blocked with 5% (w/v) non-fat milk protein in TBS-T (0.05% Tween) for 1 hour at RT. Membranes were incubated in 1 μg/mL of Fab D18 in 1× PBS for 2 hours at RT; before incubation for 1 hour, in the secondary antibody, goat-anti-human HRP-conjugated (Pierce), at 1:5000 diluted in 5% (w/v) non-fat milk protein in TBS-T. After several washes, the signal was detected using ECL kit (Amersham Pharmacia) on ECL Hypermax films (Amersham Pharmacia).

### Western blot assay

An amount equal to 25 μg of total proteins was separated by 12% SDS-PAGE and transferred to PVD membranes (Millipore). These were then blocked in 5% non-fat dried milk in TBS-T (0.05% Tween) for 1 hour at RT before incubation overnight at 4°C with primary antibody (1:1,000 or 1:2,000) diluted in 5% non-fat dried milk or 5% bovine serum albumin. After 3 washes in TBS-T, the membranes were incubated for 1 hour at RT in secondary antibody, goat-anti-mouse or rabbit, HRP-conjugated (1:2,000) diluted in 5% non-fat dried milk in TBS-T. The chemiluminescent signal was detected using ECL kit (Amersham Pharmacia) on ECL Hypermax films (Amersham Pharmacia). Densitometric analysis was performed using a Molecular Imager ChemiDoc XRS System equipped with Quantity One software (Biorad).

### Thiazolyl blue tetrazolium bromide (MTT) cell growth assay

ScGT1 and GT1 cells were treated with quinacrine (1 μM) and Fab D18 (50 nM) for 6 days. Then, about 30,000 cells per well were cultured, again in the presence of the two compounds at the same concentrations in a 96-well, tissue culture-treated plate for 4 days, measuring cell proliferation each day. To study the effects of the MEK inhibitor U0126 (Promega) on cell growth, ScGT1 and GT1 cells were seeded in a 96-well, tissue culture-treated plate and treated with U0126 (10 μM) diluted in dimethyl sulfoxide (DMSO) for 3 days. DMSO alone was used as control. In all conditions tested, every 24 hours the medium was removed and the cells were incubated with 150 μL of MTT (Sigma) working solution (0.5 mg/mL of MTT in sterile PBS) for 2 hours at 37°C. The solution was removed and formazan was solubilized adding 150 μL of DMSO to each well. The optical density was read at 560 nm and the background subtracted at 670 nm using a VersaMax plate reader (Molecular Device). Growth rate was calculated dividing the absorbance value of the last day with the value measured on the first.

### Immunofluorescence assay

Treated and untreated cells were grown overnight on glass coverslips coated with poly-L-lysine (10 μg/mL) before fixation in paraformaldehyde 4% in PBS for 20 min at RT. The cells were blocked for 1 hour at RT, in 10% normal goat serum (VECTOR Laboratories) diluted in PBS with 0.3% Triton X-100. After blocking, cells were incubated at 4°C overnight with primary antibody in dilution buffer (1% bovine serum albumin in PBS with 0.3% Triton X-100). Coverslips were washed 2 times in PBS and given an additional third washing in high-salt PBS for 2 min to decrease the aspecific binding of the antibody. After an additional wash with PBS alone, cells were further incubated for 1 hour at RT in the dark with secondary antibody, conjugated with AlexaFluor 488 (1:500; Invitrogen) in dilution buffer. Cells were further washed as described above, before mounting on Vectashield with DAPI (VECTOR Laboratories). Images were acquired with a DMIR2 confocal microscope equipped with Leica Confocal Software (Leica).

### Fluorescence quantification

In order to quantify the fluorescence in the cytosolic and nuclear compartments, random fields for each tested condition were taken at the same magnification (40×, zoom 4×). Then, a region of interest (ROI) with an area of 144 pixels (12 × 12) was chosen, and the average intensity of fluorescence within the ROI was measured in the nucleus and cytosol of every cell present in the field. More than 100 cells for every condition were analyzed. The values obtained were averaged, and the ratios between the mean value for the cytosol and the mean value for the nucleus were plotted in a histogram graph. Images were analyzed with ImageJ open source software http://rsbweb.nih.gov/ij/index.html. The results were obtained from three independent experiments.

### Statistical analysis

Student's t test was used to determine significant differences among the src family, MEK 1/2, ERK 1/2 phosphorylation, among prion infected and uninfected, and between Fab D18 treated and quinacrine treated cell lines. The levels of phosphorylation for every protein were normalized to the total amount of the same protein. All data are expressed as mean value ± SD and the values of controls are adjusted to 1; each resulting value was determined by averaging three independent experiments.

## Abbreviations used

PrP: prion protein; PrP^C^: cellular form of PrP; PrP^Sc^: scrapie isoform of PrP; N2a: mouse neuroblastoma cell line; ScN2a: scrapie-infected N2a; GT1: mouse hypothalamic cell line; ScGT1: scrapie-infected GT1; PK: proteinase K; MAPK: mitogen activated protein kinase; MEK: MAP and ERK kinases; ERK: extracellular regulated kinase.

## Competing interests

The authors declare that they have no competing interests.

## Authors' contributions

AD designed, carried out all the experiments and drafted the manuscript. GL designed the study and drafted the manuscript. All authors read and approved the manuscript.

## Supplementary Material

Additional file 1 **MEK inhibitor U0126 has no effect on cell proliferation**. ScGt1 and GT1 cells were treated for 3 days with MEK inhibitor (10 μM in DMSO) and its effects on cell proliferation were evaluated by means of MTT assay. The inhibitory effect on MEK phosphorylation at the concentration used was tested by Western blot (A). A complete inhibition of MEK phosphorylation was detected already after 1 hour of treatment with U0126 (10 μM) both in ScGT1 and GT1. The treatment conducted over 3 days on infected and uninfected cells had no statistically significant effect on cell proliferation (B). Cell growth rate was calculated as described in the Materials and Methods section. All data come from three independent experiments performed each one in 5 replicates; they are expressed as mean value ± SD. Click here for file
