# Supplementary figures and images for: Aberrant ERK 1/2 complex activation and localization in scrapie-infected GT1-1 cells
Source: Mol Neurodegener. 2010 Aug 9;5:29. doi: 10.1186/1750-1326-5-29 (PMC2928767; doi:10.1186/1750-1326-5-29)

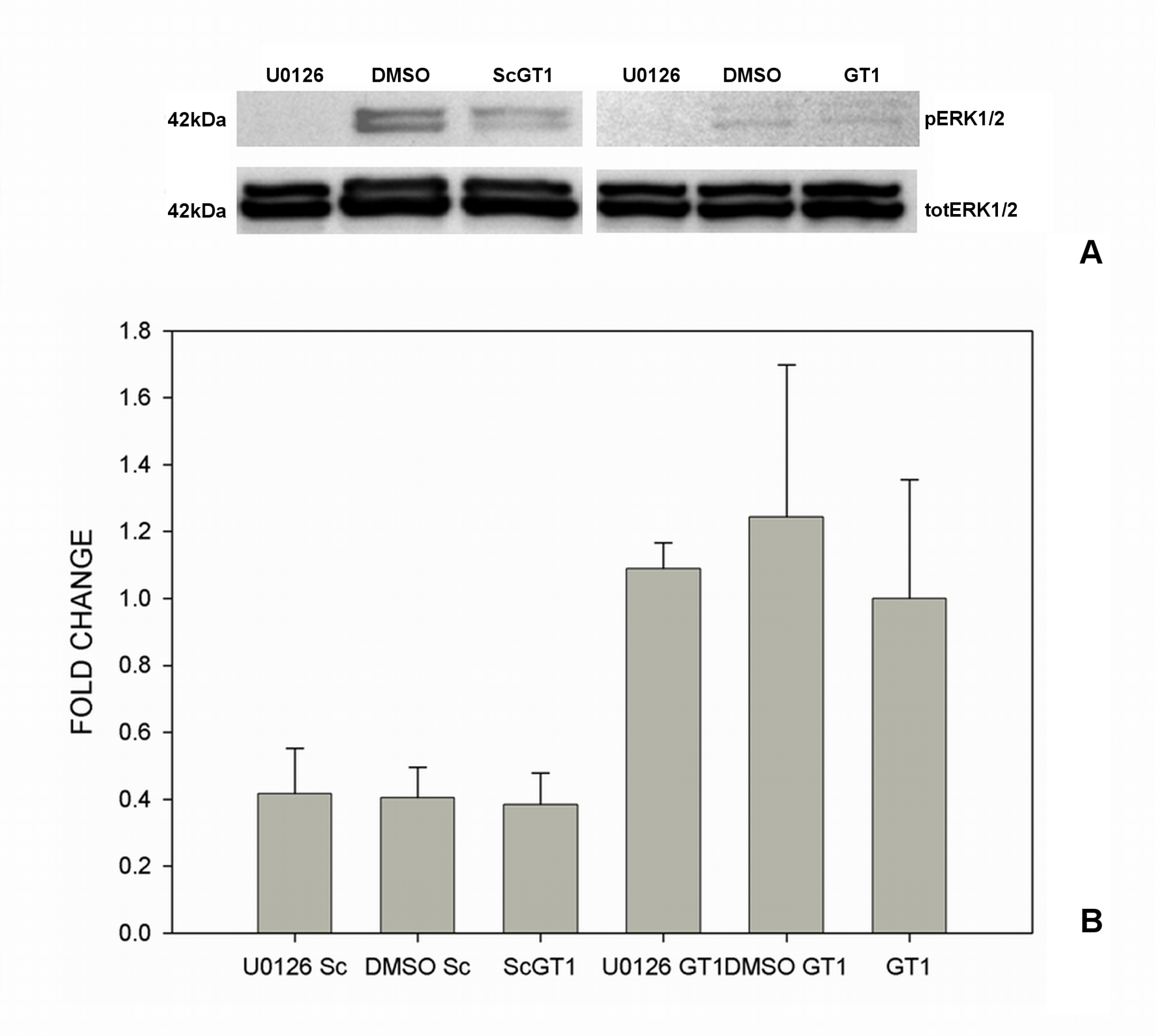

Supplement: Additional file 1 — MEK inhibitor U0126 has no effect on cell proliferation. ScGt1 and GT1 cells were treated for 3 days with MEK inhibitor (10 μM in DMSO) and its effects on cell proliferation were evaluated by means of MTT assay. The inhibitory effect on MEK phosphorylation at the concentration used was tested by Western blot (A). A complete inhibition of MEK phosphorylation was detected already after 1 hour of treatment with U0126 (10 μM) both in ScGT1 and GT1. The treatment conducted over 3 days on infected and uninfected cells had no statistically significant effect on cell proliferation (B). Cell growth rate was calculated as described in the Materials and Methods section. All data come from three independent experiments performed each one in 5 replicates; they are expressed as mean value ± SD. [file 1750-1326-5-29-S1.TIFF]
